# Risk factors associated with an outbreak of equine coronavirus at a large farm in North Carolina

**DOI:** 10.3389/fvets.2023.1060759

**Published:** 2023-03-03

**Authors:** Kate L. Hepworth-Warren, Sara J. Erwin, Caroline B. Moore, James R. Talbot, Kimberly A. S. Young, Michael J. Neault, Jennifer C. Haugland, James B. Robertson, Anthony T. Blikslager

**Affiliations:** ^1^Department of Clinical Sciences, College of Veterinary Medicine, North Carolina State University, Raleigh, NC, United States; ^2^Carolina Equine Hospital, Browns Summit, NC, United States; ^3^North Carolina Department of Agricultures and Consumer Services, Raleigh, NC, United States; ^4^Rollins Animal Disease Diagnostic Laboratory, North Carolina Department of Agriculture and Consumer Services, Raleigh, NC, United States; ^5^North Carolina Veterinary Diagnostic Laboratory System, North Carolina Department of Agriculture and Consumer Services, Raleigh, NC, United States

**Keywords:** ECoV, coronavirus, colic, diarrhea, small colon impaction

## Abstract

**Background:**

Equine coronavirus (ECoV) leads to outbreaks with variable morbidity and mortality. Few previous reports of risk factors for infection are available in the literature.

**Objectives:**

To describe unique clinical findings and risk factors for infection and development of clinical disease.

**Animals:**

135 horses on a farm affected by ECoV outbreak.

**Methods:**

Retrospective cohort study. Data obtained included age, breed, gender, activity level, housing, and feed at the onset of the outbreak. Factors were evaluated for assessment of risk of infection using simple logistic regression or Fisher's exact test. Significance was set at *p* ≤ 0.05.

**Results and findings:**

Forty-three of 54 (79.6%) horses tested on the farm were positive on fecal PCR for ECoV, and 17 horses (12.6%) developed clinical signs consistent with ECoV. Out of 17 horses in which the presence or absence of signs of colic was noted, 6 of 17 (35.3%) showed signs of colic. Three of these horses had small colon impactions, 2 of which required surgical intervention. Significant risk factors for having positive PCR results included being primarily stalled (OR 167.1, 95% CI 26.4–1719), housing next to a positive horse (OR 7.5, 95% CI 3.1–19.0), being in work (OR 26.9, 95% CI 4.6–281.9), being fed rationed hay vs. *ad libitum* (OR 1,558, 95% CI 130.8–15,593), and being fed alfalfa hay (OR 1,558, 95% CI 130.8–15,593).

**Conclusions and clinical importance:**

This report describes risk factors for ECoV infection many of which were associated with intensive management of show horses. Clinicians should be aware that clinical signs vary and can include severe colic.

## Introduction

Equine coronavirus (ECoV) is a single-stranded, positive-sense enveloped RNA virus in the *Betacoronavirus* genus. Transmission occurs primarily *via* the fecal-oral route, though viral shedding in nasal secretions has been documented in a small number of horses (1). Infection most frequently causes anorexia, fever and lethargy, but diarrhea, colic, and neurological signs have also been reported in a number of outbreaks (2–6).

At the time of publication, findings from 7 ECoV outbreaks in North America have been reported, as well as a number of others in Japan and Switzerland (2–7). These outbreaks have primarily involved adult horses, though ECoV has also been identified as a pathogen in foals with diarrhea (6, 8, 9). The morbidity in prior studies has ranged from 17 to 100% and mortality has ranged from 0 to 27% (2–5). No previous studies have included an in-depth assessment of the role of management and diet in ECoV outbreaks in horses, nor have they occurred on farms of the size described in this report (135 horses).

The objectives of this study were to describe the unique clinical and epidemiological findings associated with an ECoV outbreak that occurred on a large farm in North Carolina between February and March of 2021. Dietary and management factors associated with testing positive for ECoV on fecal PCR were assessed.

## Materials and methods

This was a retrospective cohort study focused on an ECoV outbreak that occurred between February and March of 2021 in North Carolina by individuals from the North Carolina Department of Agriculture and Consumer Services, Carolina Equine Hospital, and North Carolina State University. Data was collected retrospectively for the purpose of performing risk analyses. Data obtained for each horse on the property included the age, breed, sex, activity level, housing (stalled vs. pasture-kept), type of hay, method of feeding hay (*ad libitum* vs. ration), type of grain (based on label information) and feed at the time that the outbreak began in early February of 2021. Additional clinical information was extracted from the medical records of each horse affected by the outbreak, including date of onset of signs, presenting clinical signs, clinicopathologic data, results of fecal diagnostic testing, therapy, and outcome. Fecal PCR testing was performed at multiple laboratories, but only results coming from the lab at which the majority of horses were tested (Animal Health Diagnostic Center at Cornell University) were utilized in this study to maintain consistency (2). Additional diagnostics and treatments were performed at the discretion of the attending clinician for each case.

### Statistical analysis

GraphPad Prism software (version 9.3.1) was utilized for all statistical analyses except factor analysis. Descriptive statistics were assessed for all variables of interest. Univariate analysis was performed to assess the association of selected factors for the presence of a positive fecal ECoV PCR test. Univariate factors assessed included age, weight, sex, whether the horse was pasture-kept or stalled, whether the horse was stalled next to a positive horse, type and amount of hay fed, whether the horse was in work or not, type of concentrate fed, and the dietary starch content of the concentrate fed. Univariate analyses were completed using simple logistic regression or Fisher's exact tests when appropriate. Odds ratios and 95% confidence intervals were calculated from the maximum likelihood coefficients. Univariate factors with a significant odds ratio that did not include 1 (*p* ≤ 0.05) were used to perform multiple logistic regression. A forward stepwise approach was used, starting with univariate variables that had the highest and significant odds ratios.

R statistical software version 4.2.1 with package *FactoMineR* was used to apply factor analysis of mixed data (FAMD) to the data collected. For the sake of FAMD, data were subset to only cases which had complete information (77 cases) (10, 11).

## Results

### Patient demographics and management

**Figure 1:**
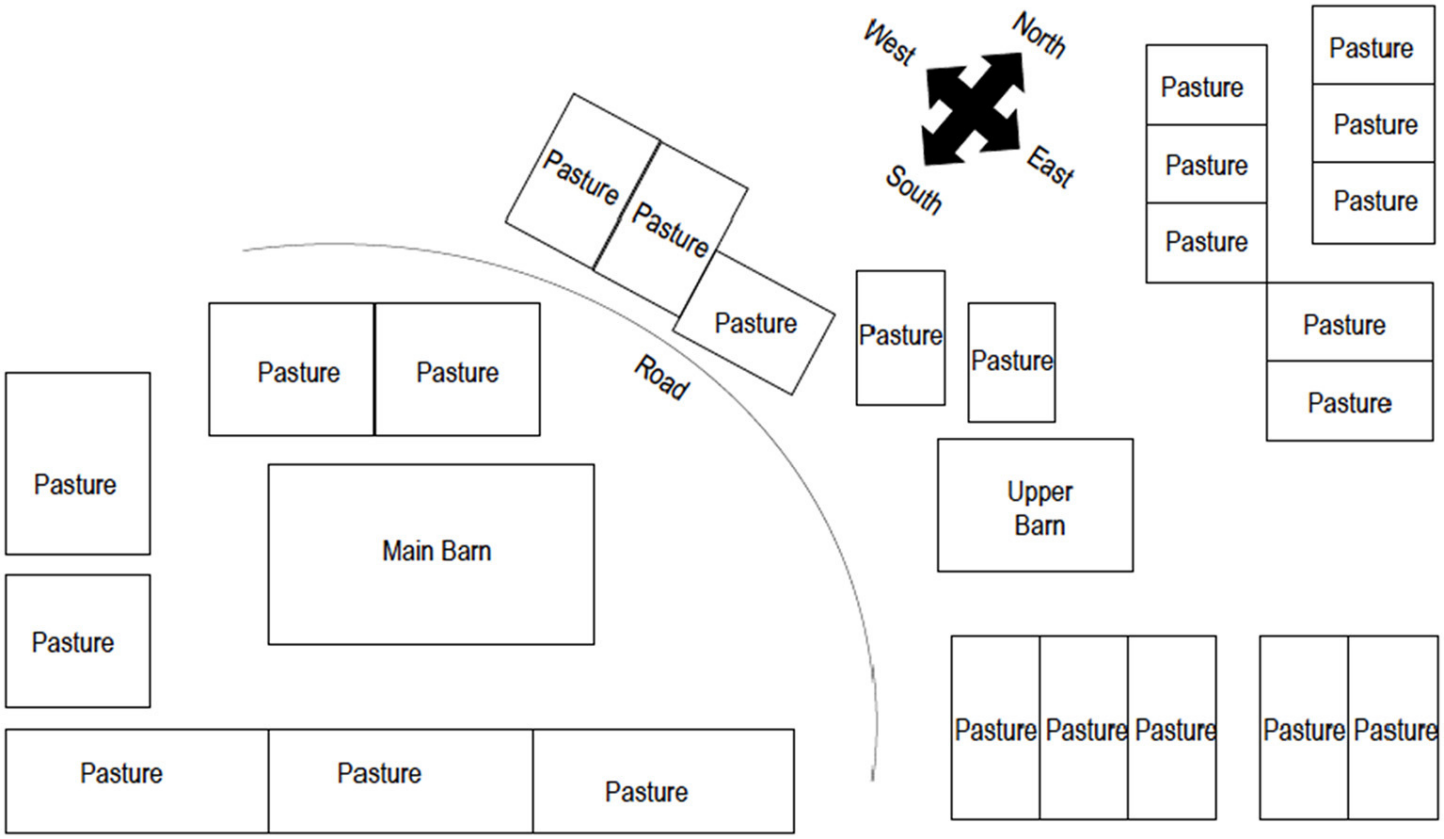
Map of farm. Drawing courtesy of Spencer Williams.

Data were collected from all 135 horses on the farm. Due to the large number of horses and the retrospective nature of the study, some patients had incomplete data. Known ages ranged from 1 to 28 years of age, with a median age of 13 years. Sex was documented for 91 horses which included 3 stallions, 43 mares, and 45 geldings. Fifty-eight of 135 horses (43.0%) were primarily stalled, and 73 of 135 (54%) horses were pasture-kept. Housing was not recorded for 4 (3%) horses. Horses were housed in groups based on their use, including horses in training, lesson horses, broodmares, retired horses, and yearlings. Specific use was available for 128 of 135 horses. The training group consisted of 46 horses, the broodmare group had 17 horses, the lesson horse group had 42 horses, and the retired horse and yearling group contained 23 horses. All horses in the training group were housed in the main barn or lower barn (Figure 1), with no outdoor turnout. The majority of lesson horses were housed in the pastures surrounding the main barn, and the majority of broodmares, retired horses and yearlings were housed in pastures on the opposite side of the road from the main barn. All horses that were in work were exercised in a shared arena. Specific management and diet information by clinical group (PCR positive and clinically affected) and by management subgroup are described in Tables 1, 2, respectively. Five different types of concentrate feed were fed on the farm. The nutritional content of each feed was reported in Table 3.

**Table 1:** Management and feeding practices for the entire farm reported in number of horses and % of population based on clinical group (PCR positive or clinically affected).

		**Total number of horses, *n* (%)**	**Total number of PCR positive horses, *n* (%)**	**Total number of clinically affected horses, *n* (%)**
Horses per group		135	42	17
**Housing**				
	Housed inside	58 (43%)	41 (97.6%)	17 (100%)
	Housed outside	73 (54%)	1 (2.4%)	0 (0%)
	Unknown	4 (3%)	0 (0%)	0 (0%)
**Type of hay**				
	Alfalfa	43 (32.3%)	41 (100%)	12 (70.6%)
	Orchard grass	78 (57.9%)	1 (0.02%)	1 (5.9%)
	Unknown	14 (9.8%)	0 (0%)	4 (23.5%)
**Hay feeding**				
	*Ad libitum*	78 (57.9%)	1 (2.4%)	1 (5.9%)
	Ration	43 (32.3%)	41 (97.6%)	12 (70.6%)
	Unknown	14 (9.8%)	0 (0%)	4 (23.5%)
**Activity level**				
	None	39 (28.9%)	1 (2.4%)	1 (5.9%)
	Light	3 (2.2%)	0 (0%)	0 (0%)
	Moderate	80 (59.3%)	34 (81.0%)	16 (94.1%)
	Unknown	13 (9.6%)	7 (16.7%)	0 (0%)
**Levothyroxine supplementation**				
	Yes	23 (17.0%)	16 (38.1%)	5 (29.4%)
	No	112 (83.0%)	26 (61.9%)	12 (70.6%)
	Unknown	0 (0%)	0 (0%)	0 (0%)
**Type of feed**				
	A	29 (21.5%)	0 (0%)	0 (0%)
	B	33 (24.4%)	0 (0%)	0 (0%)
	C	50 (37.0%)	39 (92.9%)	17 (100%)
	D	7 (5.2%)	0 (0%)	0 (0%)
	E	5 (3.7%)	0 (0%)	0 (0%)
	No grain	2 (1.5%)	0 (0%)	0 (0%)
	Unknown	9 (6.7%)	3 (4.8%)	0 (0%)

Total numbers vary due to some missing data.

**Table 2:** Management and feeding practices for farm by subgroup.

		**Total number of horses, *n* (%)**	**Training group, *n* (%)**	**Broodmare group, *n* (%)**	**Lesson horse group, *n* (%)**	**Retired and yearling group, *n* (%)**	**Unknown use, *n* (%)**
Horses per group		135	46	17	42	23	7
**Housing**							
	Housed inside	58 (43%)	46 (100%)	3 (17.6%)	4 (9.5%)	0 (0%)	5 (71.4%)
	Housed outside	73 (54%)	0 (0%)	14 (82.4%)	38 (90.5%)	20 (87.0%)	1 (14.3%)
	Unknown	4 (3%)	0 (0%)	0 (0%)	0 (0%)	3 (13.0%)	1 (14.3%)
**Type of hay**							
	Alfalfa	43 (32.3%)	41 (89.1%)	2 (11.7%)	0 (0%)	0 (0%)	0 (0%)
	Orchard grass	78 (57.9%)	0 (0%)	15 (88.2%)	41 (97.6%)	22 (95.7%)	0 (0%)
	Unknown	14 (9.8%)	5 (10.9%)	0 (0%)	1 (2.4%)	1 (4.3%)	7 (100%)
**Hay feeding**							
	*Ad libitum*	78 (57.9%)	0 (0%)	15 (88.2%)	41 (97.6%)	22 (95.7%)	0 (0%)
	Ration	43 (32.3%)	41 (89.1%)	2 (11.7%)	0 (0%)	0 (0%)	0 (0%)
	Unknown	14 (9.8%)	5 (10.9%)	0 (0%)	1 (2.4%)	1 (4.3%)	7 (100%)
**Activity level**							
	None	39 (28.9%)	0 (0%)	15 (88.2%)	2 (4.8%)	22 (95.7%)	0 (0%)
	Light	3 (2.2%)	0 (0%)	0 (0%)	3 (7.1%)	0 (0%)	0 (0%)
	Moderate	80 (59.3%)	41 (89.1%)	2 (11.7%)	37 (88.1%)	0 (0%)	0 (0%)
	Unknown	13 (9.6%)	5 (10.9%)	0 (0%)	0 (0%)	1 (4.3%)	7 (100%)
**Levothyroxine supplementation**							
	Yes	23 (17.0%)	23 (50%)	0 (0%)	0 (0%)	0 (0%)	0 (0%)
	No	112 (83.0%)	23 (50%)	17(100%)	42(100%)	23 (100%)	7 (100%)
	Unknown	0 (0%)	0 (0%)	0 (0%)	0 (0%)	0 (0%)	0 (0%)
**Type of feed**							
	A	29 (21.5%)	0 (0%)	4 (23.5%)	22 (52.4%)	3 (13.0%)	0 (0%)
	B	33 (24.4%)	0 (0%)	4 (23.5%)	20 (47.6%)	9 (39.1%)	0 (0%)
	C	50 (37.0%)	46 (100%)	4 (23.5%)	0 (0%)	0 (0%)	0 (0%)
	D	7 (5.2%)	0 (0%)	3 (17.6%)	0 (0%)	4 (17.4%)	0 (0%)
	E	5 (3.7%)	0 (0%)	2 (11.7%)	0 (0%)	3 (13.0%)	0 (0%)
	No grain	2 (1.5%)	0 (0%)	0 (0%)	0 (0%)	2 (8.7%)	0 (0%)
	Unknown	9 (6.7%)	0 (0%)	0 (0%)	0 (0%)	2 (8.7%)	7 (100%)
**PCR results**							
	Positive	42 (31.1%)	37 (80.4%)	2 (11.8%)	0 (0%)	0 (0%)	3 (42.9%)
	Negative	12 (8.9%)	9 (19.6%)	1 (5.9%)	0 (0%)	0 (0%)	2 (28.6%)
	Not tested	81 (60.0%)	0 (0%)	14 (82.4%)	42 (100%)	23 (100%)	2 (28.6%)

**Table 3:** Nutritional information from each feed.

**Type of feed**	**Crude protein %**	**Crude fiber %**	**Crude fat %**	**Dietary starch %**
A	11.5	2.0	12.0	29.0
B	14.0	4.5	11.0	15.3
C	9.5	2.0	12.5	29.5
D	13.5	5.0	10.1	29.5
E	10.5	5.5	22.5	11.5

### Timeline of outbreak

**Figure 2:**
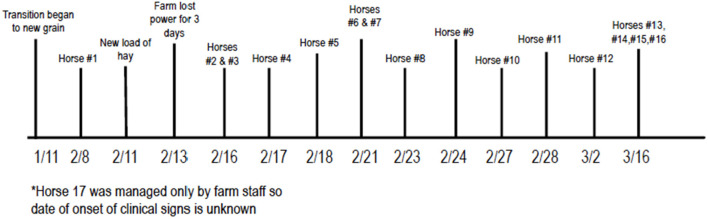
Chronologic order of events in outbreak. *Horse 17 was managed only by farm staff so date of onset of clinical signs is unknown.

On January 11th 2021, the farm began transitioning an unknown number of horses on the farm to a new grain. The first horse developed clinical signs of lethargy and diarrhea on February 8th, 2021. On February 13th, the farm lost power from the electrical grid, so all water pumps were powered by a generator for 3 days. Cases 2 and 3 then developed signs on February 16th, raising concern that the change in either grain or water may have led to initial signs. An investigation involving the North Carolina Department of Agriculture and Consumer Services began at that time to rule out toxins in the feed or water as the source of illness. Over time, 17 of 135 (12.6%) horses on the farm developed clinical signs that were ultimately attributed to ECoV (a complete timeline can be seen in Figure 2). Sixteen of these 17 (94.1%) were positive on fecal PCR for ECoV, the 17th horse was not tested due to financial constraints. This horse was also housed separately (pastured) from the rest of the affected animals.

### Diagnostic testing results

Sixteen of the 17 horses that developed clinical signs (*n* = 17) consistent with ECoV were in the training group and were stalled in either the main barn or upper barn with the exception of one horse that was housed primarily on pasture (Figure 1). Following the onset of clinical signs and ultimate diagnosis of ECoV in the index case, all but 4 horses that were housed inside (*n* = 54) were tested for ECoV via fecal PCR through the Animal Health Diagnostic Center at Cornell University, regardless of clinical status (2). Of these 54, 42 (77.8%) were positive on at least one sample. Horses were tested a median of 1 time (range: 1–3). For horses with multiple tests, time between tests was a median of 7 days (range: 1–19). Thirteen of the 17 horses with clinical signs were evaluated by a veterinarian. The remaining four were managed by farm staff, but some data regarding clinical signs were obtained. Clinical data were either extracted from medical records or obtained from farm staff but were not available for each variable (see Table 4). The majority of horses outside of the training group were not tested for ECoV, but were monitored daily by farm staff for fevers, colic, lethargy, inappetence, diarrhea, etc.

**Table 4:** Clinical signs of affected animals (*n* = 17).

**Sign**	**Yes, *n* (%)**	**No, *n* (%)**	**Unknown, *n* (%)**
Fever	3 (17.6%)	11 (64.7%)	3 (17.6%)
Diarrhea	3 (17.6%)	11 (64.7%)	3 (17.6%)
Colic	6 (35.3%)	7 (41.2%)	4 (23.5%)
Anorexia	1 (5.9%)	12 (70.6%)	4 (23.5%)
Small colon impaction	3 (17.6%)	11 (64.7%)	3 (17.6%)

Findings from abdominal palpation per rectum were recorded in 14 horses, 3/14 (21.4%) had small colon impactions. Two of these 3 underwent exploratory celiotomy. One of the horses that underwent exploratory celiotomy was also positive on fecal PCR for *Salmonella* spp., but negative on fecal culture *Salmonella* spp., and the third horse with a small colon impaction, that did not have surgery, was positive for *Salmonella* spp. on both fecal PCR and culture. A subset of 9 additional horses, including 6 that were PCR positive for ECoV and 3 that were PCR negative; had one fecal culture each performed to investigate for trends in bacterial populations between affected and unaffected horses. None of these samples grew *Salmonella* spp. and no other significant findings were noted. Additional testing for Salmonella was not pursued by the clients due to the fact that the outbreak was beginning to resolve by the time that ECoV was identified in a number of horses.

### Risk analysis

Results of univariate analysis to identify risk factors associated with positive PCR results for EcoV are reported in Table 5. Risk factors that were significantly associated with positive fecal PCR results included age (OR 0.8, *p* = 0.0005), being housed in a stall (OR 1,558, *p* < 0.0001), being in work (OR 26.9), eating a defined ration of hay (OR 1,558, *p* < 0.0001), being fed alfalfa hay (OR 1,558, *p* < 0.0001) and being fed a grain containing >15% dietary starch (OR infinity, *p* < 0.0001). Multivariate logistic regression did not produce a model with increased ORs or significance for the univariate factors assessed.

**Table 5:** Univariate analysis identifying risk factors for being positive on fecal PCV for ECoV.

**Variable**	**Positive PCR (%)**	**OR (*P*-value)**
Age (in years)	Range 1–28	0.8 (0.0005)^*^
	Mean 12.95	
Weight (lbs)	Range 700–1,200	1.007 (0.1)
	Mean 906.6	
Female	3 (25)	1.0
Male	9 (75)	3.000 (0.13)
Pasture-kept	1 (2.4)	1.0
Stalled	41 (97.6)	1,558 (< 0.0001)^*^
Not stalled next to positive	21 (51.2)	1.0
Stalled next to positive	20 (48.8)	0.76 (0.79)
Orchard grass hay	1 (2.4)	1.0
Alfalfa	41 (97.6)	1,558 (< 0.0001)^*^
*Ad libitum* hay	1 (2.4)	1.0
Ration	41 (97.6)	1,558 (< 0.0001)^*^
Not in work	1 (2.9)	1.0
In work	34 (41.5)	26.9 (< 0.0001)^*^
Dietary starch ≤ 15%	0 (0)	1.0
Dietary starch > 15%	39 (100)	+infinity (< 0.0001)^*^

Due to the retrospective nature of this study, some patient had incomplete data sets. Asterisk indicates significance.

### Factor analysis of mixed data

**Figure 3:**
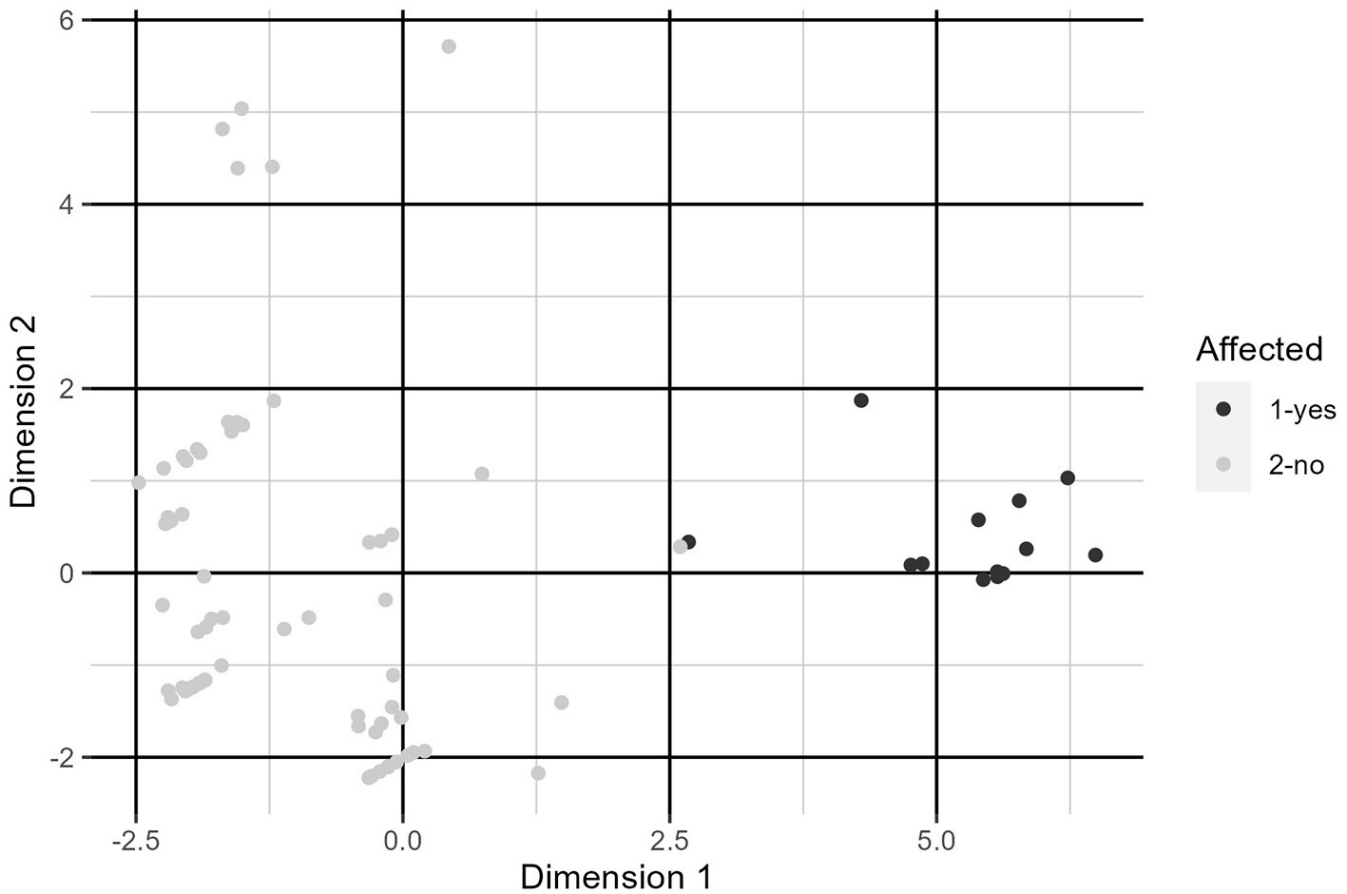
Plot of individuals along dimensions constructed by FAMD. See Table 6 for variable loadings.

Using FAMD, we were able to completely separate the clinically affected and clinically unaffected groups using a single factor which explained 32.3% of total variation in the data (Figure 3). The factor is constructed primarily from type of hay fed, amount of crude protein in the diet, patient group, housing type, whether hay was free choice, and type of feed. Exact factor loadings are given in Table 6.

**Table 6:** Factor loadings for the first two dimensions from FAMD.

	**Dim. 1**	**Dim. 2**
Age	0.199	0.038
Approximate weight	0.076	0.001
Type of hay	0.779	0.044
Activity level	0.136	0.469
Levothyroxine supplementation	0.299	0.006
Starch in diet	0.388	0.069
Crude protein	0.714	0.025
Crude fat	0.458	0.264
Crude fiber	0.008	0.451
Group	0.791	0.613
Sex	0.039	0.206
Housing	0.765	0
Stalled next to an affected horse	0.437	0
*Ad libitum* hay	0.779	0.044
Type of feed	0.912	0.788

## Discussion

Previous reports of EcoV outbreaks in North American have involved relatively small groups of horses and have not identified specific risk factors for infection (3, 4, 6). The data presented here describe unique clinical findings not previously documented in an ECoV outbreak and involved the largest number of horses of any outbreak reported in North America. Due to its size and a collaborative effort between multiple organizations the authors were able to identify a number of risk factors associated with EcoV infection.

This study describes an outbreak that took place on a farm that housed 135 horses that were managed in sub-groups based on their use, including horses in training, lesson horses, broodmares, yearlings, and retired horses. Many of the risk factors associated with infection in this outbreak were variables often associated with intensive management of performance animals, including being fed alfalfa hay (OR 1,558), being in work (OR 26.9), and being kept primarily stalled vs. pasture-kept (OR 167.1). All of the horses that tested positive on fecal PCR for ECoV were part of the training group which were kept stalled, exercised 4–5 times per week, and fed grain that reportedly contained 29.5% dietary starch. While the division of sub-groups on the farm also led to geographical separation of the horses, personnel moved between different areas of the farm and lesson horses were ridden in the same arena as horses in the training group, making it plausible that exposure to unaffected horses may still have occurred. Nonetheless, only one horse on pasture developed clinical signs of illness, though this horse was not tested so signs could not definitively be attributed to ECoV.

Feeding concentrate in addition to forage is a known risk factor for colic, and horses eating 2.5–5 kg/day and >5 kg/day of concentrate feed were at 4.8 and 6.3 times increased odds of showing signs of colic, respectively (12). The National Research Council recommend that horses receive no more than 0.2–0.4% of their body weight in dietary starch per meal, which translates to 0.9–1.8 kg per meal for an average 450 kg horse (13). While the horses in the training group on the farm described here were generally receiving quantities of feed below this recommended amount (0.65–1.96 kg of concentrate per meal), the percentage of dietary starch (29.5%) was far higher than many commercial concentrates (14–16).

High starch diets lead to a shift in the bacterial populations within the gut, which in turn can alter the immune function and make the gut more susceptible to disease (17). In healthy horses on a forage-based diet, *Fermicutes* was the most prevalent bacterial phyla with *Bacteroidetes* being the next most populous (18–20). However, in horses with undifferentiated colitis, the most prevalent phyla was *Bacteroidetes* (19). While diets that are high in starch have not induced a shift in the diversity of the microbial populations and the relative abundance of the major phyla, they lead to less stable microbial populations with higher counts of lactobacillar and streptococcal species, the latter of which has been associated with the development of laminitis in oligofructose models (21). There is mounting evidence in human medicine that alterations in the intestinal microbiome can impact viral replication an infection, and the authors speculate that alterations in the microbiome of the horses in this outbreak may have made them more susceptible to ECoV infection (22).

Anorexia, fever, and lethargy are the most frequent clinical signs in the majority of ECoV outbreaks, with colic and diarrhea reported less commonly (2–5). Due to the retrospective nature of this report, specific clinical signs were not available for all horses that were reported as being clinically affected (*n* = 17). In 14 horses in which it was documented, 6 showed signs of colic. In 2 of these 6 horses, colic signs (rolling, pawing, abdominal distention) were so severe that surgical intervention was pursued. In mice it has been documented that intestinal microbiome plays a role in viral infection, and that interactions between enteric viruses and gut flora may increase or decrease a virus' ability to replicate (23, 24). Thus, it is possible that the high starch diet of the affected horses in this study led to a shift in their microbiome that ultimately left them more susceptible to ECoV infection that other horses on the farm and potentially predisposed them to exhibiting more severe signs of colic than in previous outbreaks. While the overall fatality rate of this outbreak was 0%, the severity of colic signs was more severe than in other outbreaks (2–4).

In the 2 horses that underwent exploratory laparotomy and 1 additional horse, impactions of the small colon were identified. While small colon impactions have been associated in multiple studies with Salmonellosis, they have not been previously reported in association with ECoV infection (25, 26). One of the 2 horses that underwent exploratory laparotomy was positive for *Salmonella* spp. on fecal PCR, while the horse that did not undergo surgery was positive on both fecal culture and PCR for *Salmonella* spp. The second horse that underwent exploratory surgery was negative on two fecal samples *via* culture and PCR for Salmonella. Although signs of colic and mild large colon impactions have previously been reported in association with ECoV, severe small colon impactions requiring surgical intervention, as occurred in 2 of the horses in this study, has not been documented (7, 27). Co-infections of ECoV and *Salmonella* spp., have rarely been reported and do not have a significant association between pathogens (27, 28). In a retrospective study reporting fecal PCR testing of 3,753 fecal samples from horses > 6 months of age, there was not a significant correlation between detection of *Salmonella* and ECoV, though the exact numbers of co-infections were not reported (28). Co-infection with ECoV and either *Cryptosporidium* or *Rhodococcus equi* has been reported in neonatal foals, but not with *Salmonella* spp. (8). Hospitalized horses, especially those with systemic illness, gastrointestinal disease, or anesthesia within the 48 h prior to sampling, are known to be at a higher risk of shedding *Salmonella* spp. than healthy horses, so it is unknown whether ECoV or *Salmonella* spp. was the major cause for the signs of colic and small colon impaction in the 2 horses with Salmonellosis in this study (29). Intermittent fecal shedding of *Salmonella* spp. is known to occur, thus while 9 additional horses had single fecal cultures performed with no growth of *Salmonlla* spp. it is possible that sequential sampling may have identified the bacteria (30).

Similar to the outbreaks described by Fielding et al., there was a significant proportion of horses in this population that were positive on fecal PCR for ECoV but remained asymptomatic (4). In our study, 54 horses were tested, of which 25 were positive and asymptomatic (46.3%), similar to the 3 of 7 (43%) horses described by Fielding et al. There were an additional 80 asymptomatic horses on this farm that were not tested, so the prevalence of asymptomatic infections in this outbreak is unknown and likely higher. In previous reports of outbreaks, 4–67% of horses were positive on fecal PCR yet remained asymptomatic (2–4). An outbreak in Japan identified 26 of 41 (63%) horses in an outbreak that were asymptomatic and tested positive on virus neutralization (5). Seroprevalence (9.6% in the United States) and rates of identification of viral DNA in feces submitted to diagnostic labs (2–8.2%) are both quite low (6, 28, 31). A surveillance study investigating the prevalence of fecal shedding in 130 hospitalized horses only identified 1 out 258 samples (0.004%) that was PCR-positive for ECoV, suggesting that identification of the virus in feces is likely of clinical significance (32).

As in other reports, no horses died or were euthanized in this outbreak, though clinical signs ranged widely from minimal to severe. In the previous outbreaks described above, fatality rates ranged from 0 to 27%, though out of 7 separate outbreaks, 6 had fatality rates between 0 and 13% (2–5). Of the 17 horses that displayed clinical signs, 3 were hospitalized at a tertiary referral center, 5 were hospitalized at a secondary referral center, 5 were managed on the farm by the primary veterinarian, and the final 4 horses were managed by barn staff and not examined by a veterinarian.

Due to the retrospective nature of this study, there were a number of limitations. The close chronological association of the initial onset of clinical signs with introduction of a new batch of feed and a change in watering habits initially led to concern for toxicity which was the initial focus of diagnostics and management. The first horse to display clinical signs was also negative on fecal PCR for ECoV when first tested but was later positive. It is also plausible, given that 59.5% of the horses that had positive PCR results had subclinical infections, that other horses would have tested positive prior to the first horse that displayed clinical signs. Intermittent shedding of ECoV has also been documented, thus horses with negative PCR results may still have been infected (31). Similarly, at the time that 2 horses were taken to colic surgery, results of fecal PCR testing were not yet available, so it is unknown if those horses would ultimately have responded to medical management. Given that the horses on the farm had many different owners, testing was not performed on each horse on the farm, and therefore the total number of infected horses could have been higher. Two of the horses that were managed at a tertiary referral center were also positive on fecal PCR for *Salmonella*, one of which was also positive on culture. Nine additional horses were tested for Salmonella *via* one fecal culture, but the majority of horses were not tested. Given the known clinical similarities between Salmonellosis and ECoV, it is plausible that additional horses may have had concurrent Salmonellosis and that clinical signs in additional affected animals could have been caused by Salmonellosis (33). By the time ECoV was identified in many of the affected animals the outbreak appeared to be resolving so additional testing for other pathogens, specifically *Salmonella* spp. was not pursued. Lastly, many of the risk factors for infection identified were likely associated with animals' use as show horses. By nature, show horses are likely to be at higher risk of infection from infectious disease due to frequent transport and exposure to new populations of horses. Though exact time frames were not available, horses on this property had not attended shows in the 1–2 months prior to the outbreak, but 1–2 new horses may have been brought on to the property in the month preceding the outbreak. Many of the attempted models for the multiple logistic regression exhibited complete separation, indicating that horses that exhibited clinical symptoms and tested positive were nearly all managed in a similar way. The complete separation offered by FAMD is suggestive of a meaningful effect in the data. This requires further investigation, though, as this could be a case of overfitting to the data collected due to the sample size being small relative to the norms of the method.

The data presented here summarized a large outbreak of ECoV on a farm with clearly defined sub-populations and management strategies. The affected horses had multiple risk factors that likely contributed to them becoming infected and developing clinical signs. While additional studies are warranted to investigate the direct effect that alterations to the equine intestinal microbiome have on ECoV infection, clinicians should be aware that the combination of a high starch diet, indoor housing, and heavy work may increase the likelihood that horses become infected and develop clinical signs following exposure to ECoV.

## Data availability statement

The raw data supporting the conclusions of this article will be made available by the authors, without undue reservation.

## Ethics statement

Ethical review and approval by the Institutional Animal Care and Use Committee was not required due to the retrospective nature of this study. Written informed consent was not required or obtained from individual owners due to the retrospective nature of this study.

## Author contributions

KH-W contributed to study design, case management, data collection, and manuscript preparation. SE contributed to study design, performed the statistical analysis, and contributed to final manuscript preparation. CM and JT were heavily involved in case management and data collection. KY was involved in case management and data collection. MN and JH were involved in the data collection and the outbreak investigation. JR contributed to statistical analysis and final manuscript preparation. AB contributed to study design, statistical analysis, and manuscript preparation. All authors contributed to the article and approved the submitted version.
